# Analysis of Relationships between Immune Checkpoint and Methylase Gene Polymorphisms and Outcomes after Unrelated Bone Marrow Transplantation

**DOI:** 10.3390/cancers13112752

**Published:** 2021-06-01

**Authors:** Hidekazu Takahashi, Naoko Okayama, Natsu Yamaguchi, Moe Nomura, Yuta Miyahara, MH Mahbub, Ryosuke Hase, Yasuo Morishima, Yutaka Suehiro, Takahiro Yamasaki, Koji Tamada, Satoshi Takahashi, Arinobu Tojo, Tsuyoshi Tanabe

**Affiliations:** 1 Department of Public Health and Preventive Medicine, Yamaguchi University Graduate School of Medicine, Ube 755-8505, Japan; hidetakaha4@gmail.com (H.T.); natsu@yamaguchi-u.ac.jp (N.Y.); nomu_tym_1586@yahoo.co.jp (M.N.); hossain@yamaguchi-u.ac.jp (M.M.); hase@yamaguchi-u.ac.jp (R.H.); 2 Division of Laboratory, Yamaguchi University Hospital, Ube 755-8505, Japan; nokayama@yamaguchi-u.ac.jp (N.O.); y-miya@yamaguchi-u.ac.jp (Y.M.); t.yama@yamaguchi-u.ac.jp (T.Y.); 3 Division of Epidemiology and Prevention, Aichi Cancer Center Research Institute, Nagoya 464-8681, Japan; ymorisim@aichi-cc.jp; 4 Department of Oncology and Laboratory Medicine, Yamaguchi University Graduate School of Medicine, Ube 755-8505, Japan; ysuehiro@yamaguchi-u.ac.jp; 5 Department of Immunology, Yamaguchi University Graduate School of Medicine, Ube 755-8505, Japan; ktamada@yamaguchi-u.ac.jp; 6 Department of Hematology and Oncology, Institute of Medical Science, The University of Tokyo, Tokyo 108-8639, Japan; radius@ims.u-tokyo.ac.jp; 7 Tokyo Medical and Dental University, Tokyo 113-8510, Japan; tojo.adm@tmd.ac.jp

**Keywords:** immunoinhibitory receptor, DNA methyltransferase, histone methyltransferase, unrelated bone marrow transplantation, single-nucleotide polymorphism

## Abstract

**Simple Summary:**

Hematopoietic stem-cell transplantation (HSCT) is a curative therapy for blood disorders. Unrelated bone marrow transplantation (uBMT) is a type of allogeneic HSCT that uses the bone marrow of an unrelated donor. While HLA mismatch is a risk factor for poor outcomes in HSCT, such as graft-versus-host disease (GVHD), the importance of non-HLA single-nucleotide polymorphisms (SNPs) remains unclear. The clinical application of immune checkpoint and chromatin methylation inhibitors to cancer has been attracting attention. In the present study, we retrospectively genotyped five SNPs in four immune checkpoint genes, *BTLA*, *PD-1*, *LAG3*, and *CTLA4*, and two SNPs in methylase genes, *DNMT1* and *EZH2*, in 999 uBMT pairs. Although no correlations were observed between these SNPs and post-uBMT outcomes, recipient *EZH2* SNP exhibited a low *p*-value in the analysis of grade 2–4 acute GVHD (*p* = 0.010). This SNP may be useful for outcome predictions and needs to be confirmed in a larger-scale study.

**Abstract:**

Unrelated bone marrow transplantation (uBMT) is performed to treat blood disorders, and it uses bone marrow from an unrelated donor as the transplant source. Although the importance of HLA matching in uBMT has been established, that of other genetic factors, such as single-nucleotide polymorphisms (SNPs), remains unclear. The application of immunoinhibitory receptors as anticancer drugs has recently been attracting attention. This prompted us to examine the importance of immunoinhibitory receptor SNPs in uBMT. We retrospectively genotyped five single-nucleotide polymorphisms (SNPs) in the immune checkpoint genes, *BTLA*, *PD-1*, *LAG3*, and *CTLA4*, and two SNPs in the methylase genes, *DNMT1* and *EZH2*, in 999 uBMT donor–recipient pairs coordinated through the Japan Marrow Donor Program matched at least at HLA-A, -B, and -DRB1. No correlations were observed between these SNPs and post-uBMT outcomes (*p* > 0.005). This result questions the usefulness of these immune checkpoint gene polymorphisms for predicting post-BMT outcomes. However, the recipient *EZH2* histone methyltransferase gene SNP, which encodes the D185H substitution, exhibited a low *p*-value in regression analysis of grade 2–4 acute graft-versus-host disease (*p* = 0.010). Due to a low minor allele frequency, this SNP warrants further investigation in a larger-scale study.

## 1. Introduction

Hematopoietic stem-cell transplantation (HSCT) is a curative therapy for blood disorders, such as leukemia. Unrelated bone marrow transplantation (uBMT) is an allogeneic HSCT that uses bone marrow from an unrelated donor as the transplant source. Due to advances in treatment, overall survival (OS) after uBMT, similar to other types of allogeneic HSCTs, has been prolonged; however, further improvements are needed. Acute and chronic graft-versus-host disease (GVHD) pose serious risks to post-transplant recipients. HLA mismatches between a recipient and donor are well-established risk factors for poor HSCT outcomes [1,2,3]. Moreover, even when all 12 HLA-A, -B, -C, -DPB1, -DQB1, and -DRB1 loci are matched at allele levels, severe (grade 3–4) acute GVHD (aGVHD) occurred in 11% of recipients and the 5 year overall survival rate was 53% in uBMT [3]. However, it currently remains unclear whether genetic polymorphisms outside HLA loci affect HSCT outcomes. Non-HLA single-nucleotide polymorphisms (SNPs) are attracting interest because of their potential in outcome predictions [4], as well as donor selection. We previously investigated the relationships between SNPs in innate immune pathway genes and BMT outcomes [5,6].

Inhibitory receptors on T cells, such as cytotoxic T-lymphocyte associated-4 (CTLA4) [7], programmed cell death 1 (PD1; PDCD1) [8], B and T lymphocyte attenuator (BTLA) [9], and lymphocyte activation gene-3 (LAG-3) [10], prevent uncontrolled T-cell activation. These inhibitory receptors, particularly PD1 and CTLA4, have been attracting increasing interest because they exert anticancer effects when blocked [11,12], and they have been studied in preclinical aGVHD models [13].

Chromatin-modifying enzymes, particularly DNA and histone methylases, play important roles in the development of hematological malignancies [14]. Hypomethylating agents, such as azacytidine, are in clinical use for patients with acute myeloid leukemia (AML) and myelodysplastic syndromes (MDS) [15]. The administration of azacytidine causes the demethylation of the PD1 promoter in patients with AML and MDS [16], and it has also been shown to mitigate GVHD in mice [17]. A target of azacytidine, DNA methyltransferase 1 (DNMT1), methylates the cytosine residues of DNA to maintain the DNA methylation pattern [15,18]. The *Enhancer of Zeste 2* (*EZH2*) gene encodes histone H3 lysine 27 methyltransferase and is a component of polycomb repressive complex-2 (PRC-2) [19]. Ezh2 interacts with DNMTs and is required for the DNA methylation of Ezh2 target promoters [20]. Ezh2 appears to play important roles in hematological malignancies [21,22]. Its loss in donor T cells inhibits GVHD in mice [23]; however, this may not be the case in humans [24]. Cancer cells exploit the PRC2-mediated transcriptional silencing of the MHC-I antigen processing pathway, evading T-cell-mediated immunity [25].

To the best of our knowledge, while many candidate SNP studies have investigated the relationship between *CTLA4* and post-HSCT outcomes in humans [26,27,28,29,30,31,32,33], no or only a few candidate SNP studies have been published on the relationship between *PD1* [34], *BTLA*, *LAG3*, *DNMT1*, and *EZH2* and post-HSCT outcomes in humans.

Therefore, we herein investigated the relationships between SNPs in the four aforementioned immunoinhibitory receptors genes and two methylases genes and the outcomes of uBMT matched at least at HLA-A, -B, and -DRB1, but not necessarily at HLA-C, which is a typical HLA matching pattern in uBMT [35], coordinated between May 2006 and April 2009 through the Japan Marrow Donor Program (JMDP) [36].

## 2. Materials and Methods

### 2.1. Subjects

The characteristics and transplantation outcomes of the donors and patients in the present study were previously described (Table S1, of Takahashi et al. [6]). Briefly, 999 donor–recipient pairs who satisfied all of the following criteria were retrospectively genotyped: the pair underwent uBMT between May 2006 and April 2009 through the JMDP; Japanese ethnicity; donor age of at least 20 years; days of survival were available; HLA-A, -B, -C, -DPB1, -DQB1, and -DRB1 alleles were retyped; HLA-A, -B, and -DRB1 were confirmed to be matched (HLA-C, -DPB1, and -DQB1 were not necessarily matched) [6]. Among them, 822 malignant-disease patients without a previous transplantation history and their uBMT donors were the subjects of the main analysis (Table S1), similar to the previous study [6]. Clinical data were collected by the Japan Society for Hematopoietic Cell Transplantation using the Transplant Registry Unified Management Program (TRUMP) [37].

### 2.2. SNP Selection

We selected SNPs that encode nonsynonymous amino-acid substitutions and have a minor allele frequency (MAF) >0.05 in 104 Japanese residents of Tokyo (JPT104) from the 1000 Genomes Project [38]. We used rs9288952 and rs76844316 for *BTLA*, rs2227982 for *PDCD1*, rs870849 for *LAG3*, rs231775 for *CTLA4*, rs2302427 for *EZH2*, and rs2228612 for *DNMT1* (Tables S2 and S3, Supplementary Materials). Among these SNPs, rs76844316 and rs231775 are functional SNPs, with one of the two alleles of each SNP exhibiting stronger activity than the other allele in cultured cells (Table S2) [39,40].

### 2.3. SNP Genotyping

Genomic DNA preparation, TaqMan SNP Genotyping Assays (Applied Biosystems, Waltham, MA, USA), and direct Sanger sequencing following PCR were performed as previously described [6]. In TaqMan assays, the default threshold set by the software (quality = 95) was used for all SNPs, except rs2227982, for which quality = 90 was used as the threshold. To confirm the results of these TaqMan assays, approximately 10 samples for each of the three genotypes, two homozygous and one heterozygous, were re-genotyped by direct Sanger sequencing following PCR using the primers shown in Table S2. DNA samples unsuccessful in TaqMan genotyping were then genotyped by the aforementioned direct Sanger sequencing following PCR.

### 2.4. Outcomes

The primary outcome of the present study was grade 2–4 aGVHD within 100 days of transplantation. Secondary outcomes were grade 3–4 aGVHD within 100 days, chronic GVHD (cGVHD), extensive cGVHD (ecGVHD), OS, non-relapse mortality (NRM), and relapse. The detailed definitions and competing events of these outcomes used in the present study were identical to those previously described [6].

### 2.5. Covariates

Recipient characteristics used as covariates were sex, age, disease stage, body mass index, the cytomegalovirus (CMV) serostatus, and performance status. The donor characteristics used were sex, age, and the CMV serostatus. Transplantation characteristics used as covariates were myeloablative conditioning, cyclosporine A, arabinofuranosyl cytidine (Ara-C), cyclophosphamide, the number of nucleated cells infused, and days from diagnosis to BMT. Mismatches between a recipient and the corresponding donor of the ABO blood type, HLA-C, HLA-DPB1, and HLA-DQB1 were also used as covariates. Total HLA mismatches denote the sum of the numbers of mismatches at HLA-C, -DPB1, and -DQB1 because HLA-A, -B, and -DRB1 were matched. These covariates are shown in Table S1 and were previously described [6].

### 2.6. Statistical Analysis

Statistical analyses were conducted as previously described [6]. The additive genetic model was defined as the risk per number of the minor allele. The recessive model was defined as the risk of the minor homozygous genotype relative to the two other genotypes, and the dominant model was similarly defined. OS was examined using Cox proportional hazard regression. Other outcomes were analyzed by the Fine–Gray proportional sub-distribution hazard (SH) regression. Reported covariates fixed in multivariable regression of aGVHD, cGVHD, and OS were previously described [6]. Fixed covariates in multivariable regression of NRM and relapse were the same as those used in multivariable regression of OS. The Wald test was used to calculate *p*-values in the regression analysis. All *p*-values are two-tailed unadjusted values, and *p* < 0.005 was considered to be significant. The R software (ver. 3.2), the EZR software [41], VCFtools (ver. 0.1.11), and Microsoft Excel were used.

## 3. Results

### 3.1. Genotyping

We genotyped all SNPs in 999 subjects, except for the LAG3 SNP, rs870849. It was not possible to genotype one recipient for rs870849. The TaqMan assay produced a very weak signal and three independent trials of PCR amplification for the Sanger sequencing failed for an unknown reason; therefore, the rs870849 genotype of this recipient was finalized as undetermined (Table S3). However, this did not affect the statistical analysis because this recipient underwent a previous transplantation and was not a subject in the main analysis.

Allele frequencies were similar among the donors, recipients, and 104 Japanese individuals in Tokyo (JPT104) from the 1000 Genomes Project (Table S4). The null hypothesis for the Hardy–Weinberg equilibrium (HWE) was not rejected for any SNPs (*p* > 0.005; Table S4, Supplementary Materials). Linkage disequilibrium (LD) between two SNPs in a single gene, namely, rs9288952 and rs76844316 in BTLA, was similar among the recipients, donors, and JPT104 (Table S5). Therefore, there was no strong selection for or against a certain genotype at these SNPs in uBMT donors or recipients, and, as a consequence, all SNPs were included in the statistical analysis.

### 3.2. Grade 2–4 Acute GVHD

We conducted a univariable regression of grade 2–4 aGVHD, the primary outcome, on these seven SNPs under additive, dominant, and recessive genetic models. None of these SNPs correlated with grade 2–4 aGVHD (*p* > 0.005; Table S6). We then performed the multivariable regression analysis previously described, which is based on BIC-based covariate selection and takes interaction terms into account. However, no SNP variable or SNP–covariate interaction terms correlated with grade 2–4 aGVHD (data not shown). To make the results comparable with previous findings, we also performed a multivariable regression adjusted with reported covariates for grade 2–4 aGVHD. The results obtained were essentially unchanged, and no SNP correlated with grade 2–4 aGHVD (*p* > 0.005; Table 1). Among them, the recipient *EZH2* SNP in the additive model exhibited low *p*-values (*p* = 0.011 and 0.010 in univariable and multivariable analyses, respectively).

### 3.3. Secondary Outcomes

We performed univariable and multivariable regression analyses, similar to grade 2–4 acute GVHD, of secondary outcomes, namely, grade 3–4 aGVHD, ecGVHD, cGVHD, OS, non-recurrent mortality, and relapse (*p* > 0.005; Table 2, Table 3, Table 4, Table 5, Table 6 and Table 7 and Tables S7–S12). However, no SNPs correlated with any of these outcomes. Therefore, correlations were not observed between these immune checkpoint and methylase genes and BMT outcomes.

## 4. Discussion

In the present study, we initially examined the relationships between polymorphisms in the immune checkpoint genes, *BTLA*, *PD-1*, *LAG3*, and *CTLA4*, and BMT outcomes, and we found no correlations.

In a murine model, BTLA played distinct roles in GVHD; the administration of an anti-BTLA monoclonal antibody inhibited donor anti-host T-cell responses, whereas BTLA also served as a ligand that sent a prosurvival signal in donor T cells [42]. The two *BTLA* SNPs tested in the present study were not associated with aGVHD. One explanation for this result is a difference between mice and humans. For example, many murine BMT models are MHC-mismatched and humans do not have a genetically uniform background, which is in contrast to experimental animals. Another explanation is the nature of these two SNPs. Both SNPs are non-synonymous in terms of amino-acid substitutions. One of them, rs9288952, is associated with malignant breast cancer in Chinese women [43] and has an MAF > 0.25; however, no molecular function has been reported. Therefore, this SNP may not be a functional SNP or may be associated with a functional SNP. The other BTLA SNP, rs76844316, was shown to be functional, with the T, but not G, allele being inhibitory against concanavalin A- and anti-CD3 Ab-induced IL-2 production when retrovirally introduced into Jurkat T cells [39]. Although this SNP is associated with susceptibility to rheumatoid arthritis (RA), the MAF is <0.1. Therefore, the lack of a correlation between this SNP and aGVHD may be attributed to its low MAF. The location of a variant within the BTLA gene may also be important. These two SNPs encode amino-acid substitutions within the cytoplasmic domain of the BTLA protein (https://www.uniprot.org/uniprot/Q7Z6A9 (last accessed in 1 March 2021)). There was no SNP within the extracellular domain affecting ligand binding that met our SNP selection criteria. Collectively, the present results do not necessarily indicate the true lack of a relationship between the BTLA gene and aGVHD.

PD-1 is another immune checkpoint protein, and its blockade is of widespread interest as a treatment for cancer, including Hodgkin’s lymphoma [8,44]. Blockade of the PD-1/PD-L pathway has been shown to enhance aGVHD lethality in mice [45]. In a Spanish study, two PD-1 SNPs in donors were associated with grade 2–4 aGVHD in HLA-identical sibling HSCT [34]. In the present study, another *PD-1* SNP, rs2227982, was not associated with any post-uBMT outcomes. Although rs2227982 may not be a functional *PD-1* SNP, previous studies on east Asians reported correlations with the risk of type 1 diabetes, with glucose and insulin levels after the oral glucose tolerance test [46], and with ankylosing spondylitis [47]. Therefore, even if *PD-1* is associated with BMT outcomes, this relationship may depend on specific clinical characteristics. A similar explanation may be applied to the *LAG3* SNP used in the present study, rs870849, which was previously shown to be associated with multiple sclerosis [48].

*CTLA4* SNPs correlated with transplant outcomes in some [27,28,30,31,32], but not all HSCT studies [33,49], and discrepancies were noted in the findings obtained in the studies that reported correlations [26,29]. The *CTLA4* SNP used in the present study, rs231775, encodes an Ala-to-Thr substitution at the 17th amino-acid position; the CTLA4 protein with threonine 17 more strongly inhibits T-cell activation [40]. Despite this reported functionality, no correlation was found between this SNP and transplant outcomes in the present study. Therefore, *CTLA4* may not be associated with BMT outcomes, or, even if it is, the relationship may depend on some factors, such as patient characteristics.

We also examined the relationships between polymorphisms in the methylase genes, *DNMT1* and *EZH2*, and BMT outcomes. The *DNMT1* SNP used, rs2228612, was identified as a cancer risk factor in a meta-analysis [50]. However, no correlations were observed between this SNP and post-BMT outcomes in the present study. Although DNA methyltransferase inhibitors are used to treat AML and MDS, azacitidine inhibits not only DNMT1, but also the de novo DNA methylases, DNMT3a and DNMT3b [15]. This is one possible explanation for the lack of a relationship between the *DNMT1* SNP and post-BMT outcomes. *DNMT3a* and *DNMT3b* SNPs were not included in the present study because we did not detect any SNPs in these genes with sufficiently high MAFs in the Japanese population.

As already discussed, EZH2 is involved in the silencing of MHC-I [25], as well as in GVHD in mice [23]. The *EZH2* SNP, rs2302427, encodes the Ezh2 D185H substitution, which is located in the domain that interacts with DNA methyltransferases (Table S2). This SNP has been identified as a risk factor for colorectal cancer in a Chinese population [51]. This SNP was also detected in an AML patient with a morphology resembling acute promyelocytic leukemia without the PARA gene rearrangement [52]. In the present study, recipient rs2302427 exhibited a low *p*-value in the analysis of grade 2–4 aGVHD (*p* = 0.010) in the additive model (Table 1) despite its low MAF of 0.09 (Table S3). Therefore, recipient rs2302427 warrants further study with a larger subject size, particularly in a recessive model that was not examined in the present study due to the low MAF (Table 1). The rs2302427 recessive model will test the GG genotype, which encodes the homozygous Ezh2 H185 protein (Table S2).

The present study had a number of limitations. The study design was retrospective, and clinical decisions should be based on prospective studies. Furthermore, SNP–SNP interactions were not considered. Several of the genes analyzed may be redundant for HSCT outcomes in humans. Therefore, future studies on a larger sample size are needed. In addition, the molecular functions of five of the SNPs are unknown (Table S2).

## 5. Conclusions

Although we expected immune checkpoint gene polymorphisms to be useful for predicting BMT outcomes, no correlations were observed. The recipient *EZH2* SNP, rs2302427, which encodes the D185H substitution, exhibited a low *p*-value in analysis of grade 2–4 aGVHD despite a low MAF and, thus, warrants further investigation in a large-scale study.

## Figures and Tables

**Table 1:** Multivariable regression of grade 2–4 aGVHD on donor and recipient SNPs.

Gene	SNP (Donor/Recipient)	Additive Model		Dominant Model		Recessive Model	
		SHR (95% CI)	*p*-Value	SHR (95% CI)	*p*-Value	SHR (95% CI)	*p*-Value
*BTLA*	rs9288952 (d)	1.07 (0.89–1.28)	0.486	1.10 (0.87–1.39)	0.441	1.04 (0.68–1.60)	0.851
*BTLA*	rs76844316 (d)	1.02 (0.77–1.34)	0.893	1.08 (0.79–1.48)	0.622	N.A. ^†^	N.A.
*PDCD1*	rs2227982 (d)	0.94 (0.79–1.12)	0.493	0.90 (0.69–1.17)	0.430	0.95 (0.72–1.26)	0.728
*LAG3*	rs870849 (d)	1.00 (0.79–1.25)	0.968	1.03 (0.79–1.33)	0.850	N.A. ^†^	N.A.
*CTLA4*	rs231775 (d)	0.87 (0.73–1.03)	0.116	0.84 (0.66–1.07)	0.163	0.82 (0.58–1.15)	0.254
*EZH2*	rs2302427 (d)	0.81 (0.62–1.08)	0.147	0.88 (0.64–1.21)	0.424	N.A. ^†^	N.A.
*DNMT1*	rs2228612 (d)	0.91 (0.76–1.09)	0.313	0.93 (0.74–1.19)	0.580	0.75 (0.48–1.18)	0.215
*BTLA*	rs9288952 (r)	0.88 (0.73–1.06)	0.188	0.83 (0.66–1.06)	0.135	0.91 (0.59–1.42)	0.690
*BTLA*	rs76844316 (r)	0.88 (0.62–1.25)	0.491 ^‡^	0.84 (0.59–1.21)	0.351 ^‡^	N.A. ^†^	N.A.
*PDCD1*	rs2227982 (r)	1.00 (0.85–1.17)	0.990	1.06 (0.81–1.37)	0.674	0.94 (0.71–1.24)	0.643
*LAG3*	rs870849 (r)	0.96 (0.77–1.19)	0.711	0.97 (0.76–1.26)	0.842	N.A. ^†^	N.A.
*CTLA4*	rs231775 (r)	0.96 (0.81–1.14)	0.655	0.91 (0.71–1.15)	0.413	1.04 (0.75–1.44)	0.798
*EZH2*	rs2302427 (r)	0.64 (0.46–0.90)	0.010	0.66 (0.46–0.95)	0.026	N.A. ^†^	N.A.
*DNMT1*	rs2228612 (r)	1.07 (0.91–1.27)	0.411	1.05 (0.83–1.34)	0.662	1.19 (0.86–1.65)	0.290

The results for each SNP were obtained by running separate regressions under the three genetic models indicated in the top row of the table, adjusted with total HLA mismatches, CyA, BMI, myeloablative, disease stage, recipient age, donor age, and female donor–male recipient. In the second column of the table, (d) and (r) stand for donor and recipient, respectively. Malignant-disease patients without a previous transplantation history were analyzed (*n* = 787). Excluded: aGVHD-unevaluable (*n* = 34) and the day of grade 2/3/4 aGVHD unknown (*n* = 1). *n* of the primary competing events (grade 3–4 aGVHD) = 80. SHR, subdistribution hazard ratio; CI, confidence interval. Malignant-disease patients without a previous transplantation history were included (*n* = 787). Excluded: aGVHD-unevaluable (*n* = 34) and the day of grade 2/3/4 aGVHD unknown (*n* = 1). The number of primary competing events (grade 2–4 aGVHD) = 280. ^†^ Not applicable (N.A.) due to a low minor allele frequency. ^‡^ The proportional SHR assumption is violated (*p* for the interaction between a variable and time < 0.005). The lowest *p* in this study (*p* = 0.010) is shown in bold and was not significant (*p* > 0.005 by the Wald test).

**Table 2:** Multivariable regression of grade 3–4 aGVHD on donor and recipient SNPs.

Gene	SNP (Donor/Recipient)	Additive Model		Dominant Model		Recessive Model	
		SHR (95% CI)	*p*-Value	SHR (95% CI)	*p*-Value	SHR (95% CI)	*p*-Value
*BTLA*	rs9288952 (d)	1.13 (0.80–1.59)	0.491	1.06 (0.68–1.64)	0.799	1.52 (0.78–2.94)	0.220
*BTLA*	rs76844316 (d)	0.72 (0.40–1.28)	0.262	0.74 (0.39–1.38)	0.337	N.A. ^†^	N.A.
*PDCD1*	rs2227982 (d)	0.90 (0.67–1.22)	0.516	0.93 (0.57–1.53)	0.789	0.81 (0.47–1.39)	0.439
*LAG3*	rs870849 (d)	0.65 (0.38–1.11)	0.112	0.58 (0.33–1.01)	0.054	N.A. ^†^	N.A.
*CTLA4*	rs231775 (d)	1.36 (1.01–1.84)	0.046	1.70 (1.03–2.83)	0.040	1.28 (0.72–2.28)	0.406
*EZH2*	rs2302427 (d)	0.83 (0.48–1.42)	0.490	0.89 (0.48–1.65)	0.708	N.A. ^†^	N.A.
*DNMT1*	rs2228612 (d)	1.09 (0.78–1.51)	0.615	1.20 (0.75–1.91)	0.443	0.92 (0.43–1.97)	0.836
*BTLA*	rs9288952 (r)	0.89 (0.63–1.26)	0.524	0.90 (0.58–1.41)	0.644	0.75 (0.30–1.86)	0.531
*BTLA*	rs76844316 (r)	0.86 (0.42–1.75)	0.674	0.70 (0.35–1.43)	0.334	N.A. ^†^	N.A.
*PDCD1*	rs2227982 (r)	0.91 (0.68–1.23)	0.543	0.96 (0.59–1.57)	0.885	0.79 (0.45–1.38)	0.404
*LAG3*	rs870849 (r)	0.84 (0.56–1.25)	0.385	0.88 (0.55–1.43)	0.618	N.A. ^†^	N.A.
*CTLA4*	rs231775 (r)	1.00 (0.73–1.38)	0.983	1.01 (0.64–1.59)	0.969	1.00 (0.53–1.86)	0.992
*EZH2*	rs2302427 (r)	0.72 (0.39–1.33)	0.294	0.74 (0.38–1.45)	0.383	N.A. ^†^	N.A.
*DNMT1*	rs2228612 (r)	1.16 (0.84–1.62)	0.360	1.11 (0.71–1.73)	0.659	1.47 (0.82–2.65)	0.195

The results for each SNP were obtained by running separate regressions under the three genetic models indicated in the top row of the table, adjusted with total HLA mismatches, CyA, BMI, myeloablative, disease stage, recipient age, donor age, and female donor–male recipient. Malignant-disease patients without a previous transplantation history were analyzed (*n* = 787). Excluded: aGVHD-unevaluable (*n* = 34) and the day of grade 2/3/4 aGVHD unknown (*n* = 1). The number of primary competing events (grade 3–4 aGVHD) = 80. ^†^ Not applicable (N.A.) due to a low minor allele frequency. See the legend of Table 1 for other notations.

**Table 3:** Multivariable regression of extensive chronic GVHD on donor and recipient SNPs.

Gene	SNP (Donor/Recipient)	Additive Model		Dominant Model		Recessive Model	
		SHR (95% CI)	*p*-Value	SHR (95% CI)	*p*-Value	SHR (95% CI)	*p*-Value
*BTLA*	rs9288952 (d)	0.94 (0.71–1.24)	0.657	0.89 (0.63–1.27)	0.527	1.04 (0.57–1.89)	0.894
*BTLA*	rs76844316 (d)	0.91 (0.61–1.35)	0.629	0.97 (0.62–1.52)	0.899	N.A. ^†^	N.A.
*PDCD1*	rs2227982 (d)	1.10 (0.84–1.44)	0.475	0.98 (0.65–1.48)	0.929	1.28 (0.87–1.89)	0.205
*LAG3*	rs870849 (d)	0.88 (0.61–1.27)	0.481	0.86 (0.58–1.29)	0.470	N.A. ^†^	N.A.
*CTLA4*	rs231775 (d)	0.97 (0.76–1.25)	0.835	0.96 (0.67–1.37)	0.809	0.98 (0.61–1.59)	0.942
*EZH2*	rs2302427 (d)	0.64 (0.39–1.05)	0.079	0.60 (0.36–1.02)	0.060	N.A. ^†^	N.A.
*DNMT1*	rs2228612 (d)	1.07 (0.82–1.41)	0.606	1.01 (0.72–1.43)	0.935	1.32 (0.77–2.27)	0.311
*BTLA*	rs9288952 (r)	0.90 (0.69–1.18)	0.443	0.86 (0.61–1.22)	0.392	0.93 (0.50–1.70)	0.806
*BTLA*	rs76844316 (r)	1.31 (0.85–2.04)	0.221	1.26 (0.80–1.99)	0.309	N.A. ^†^	N.A.
*PDCD1*	rs2227982 (r)	0.93 (0.74–1.18)	0.553	0.95 (0.65–1.38)	0.770	0.87 (0.57–1.32)	0.498
*LAG3*	rs870849 (r)	0.76 (0.53–1.10)	0.153	0.71 (0.47–1.05)	0.089	N.A. ^†^	N.A.
*CTLA4*	rs231775 (r)	0.89 (0.69–1.15)	0.373	0.87 (0.61–1.24)	0.453	0.83 (0.49–1.40)	0.478
*EZH2*	rs2302427 (r)	1.10 (0.71–1.71)	0.679	1.05 (0.66–1.66)	0.847	N.A. ^†^	N.A.
*DNMT1*	rs2228612 (r)	0.96 (0.75–1.24)	0.780	0.97 (0.69–1.37)	0.865	0.92 (0.54–1.57)	0.752

The results for each SNP were obtained by running separate regressions under the three genetic models indicated in the top row of the table, adjusted with HLA-C mismatch, BMI, myeloablative, disease stage, recipient age, donor age, and female donor–male recipient. Malignant-disease patients without a previous transplantation history were analyzed (*n* = 677). Excluded: cGVHD-unevaluable (*n* = 142) and the day of cGVHD unknown (*n* = 3). The number of primary competing events (ecGVHD) = 132. ^†^ Not applicable (N.A.) due to a low minor allele frequency. See the legend of Table 1 for other notations. No SNP correlated with ecGVHD (*p* > 0.005 by the Wald test).

**Table 4:** Multivariable regression of all chronic GVHD on donor and recipient SNPs.

Gene	SNP (Donor/Recipient)	Additive Model		Dominant Model		Recessive Model	
		SHR (95% CI)	*p*-Value	SHR (95% CI)	*p*-Value	SHR (95% CI)	*p*-Value
*BTLA*	rs9288952 (d)	0.94 (0.77–1.14)	0.513	0.93 (0.72–1.21)	0.605	0.87 (0.54–1.41)	0.568
*BTLA*	rs76844316 (d)	1.12 (0.85–1.50)	0.418	1.19 (0.86–1.64)	0.289	N.A. ^†^	N.A.
*PDCD1*	rs2227982 (d)	0.98 (0.81–1.18)	0.837	0.95 (0.70–1.28)	0.723	1.00 (0.74–1.35)	0.993
*LAG3*	rs870849 (d)	0.97 (0.74–1.27)	0.819	0.89 (0.66–1.20)	0.441	N.A. ^†^	N.A.
*CTLA4*	rs231775 (d)	1.04 (0.86–1.25)	0.690	1.00 (0.77–1.31)	0.985	1.14 (0.82–1.59)	0.442
*EZH2*	rs2302427 (d)	0.71 (0.51–1.00)	0.053	0.72 (0.49–1.05)	0.087	N.A. ^†^	N.A.
*DNMT1*	rs2228612 (d)	1.02 (0.84–1.25)	0.832	1.04 (0.80–1.35)	0.766	0.99 (0.63–1.56)	0.977
*BTLA*	rs9288952 (r)	0.99 (0.81–1.20)	0.880	1.02 (0.79–1.32)	0.880	0.86 (0.53–1.39)	0.531
*BTLA*	rs76844316 (r)	1.26 (0.90–1.77)	0.177	1.27 (0.89–1.81)	0.186	N.A. ^†^	N.A.
*PDCD1*	rs2227982 (r)	0.94 (0.78–1.13)	0.483	0.86 (0.65–1.13)	0.269	0.99 (0.73–1.35)	0.953
*LAG3*	rs870849 (r)	0.93 (0.73–1.19)	0.587	0.89 (0.67–1.18)	0.413	N.A. ^†^	N.A.
*CTLA4*	rs231775 (r)	0.89 (0.73–1.07)	0.200	0.90 (0.69–1.17)	0.434	0.75 (0.49–1.13)	0.165
*EZH2*	rs2302427 (r)	0.95 (0.68–1.33)	0.763	0.94 (0.65–1.36)	0.749	N.A. ^†^	N.A.
*DNMT1*	rs2228612 (r)	0.90 (0.74–1.09)	0.265	0.87 (0.68–1.13)	0.298	0.85 (0.56–1.30)	0.460

The results for each SNP were obtained by running separate regressions under the three genetic models indicated in the top row of the table, adjusted with HLA-C mismatch, BMI, myeloablative, disease stage, recipient age, donor age, and female donor–male recipient. Malignant-disease patients without a previous transplantation history were analyzed (*n* = 677). Excluded: cGVHD-unevaluable (*n* = 142) and the day of cGVHD unknown (*n* = 3). The number of primary competing events (limited + extensive cGVHD) = 235. ^†^ Not applicable (N.A.) due to a low minor allele frequency. See the legend of Table 1 for other notations. No SNP correlated with all cGVHD (*p* > 0.005 by the Wald test).

**Table 5:** Multivariable Cox regression of overall survival (OS) on donor and recipient SNPs.

Gene	SNP (Donor/Recipient)	Additive Model		Dominant Model		Recessive Model	
		HR (95% CI)	*p*-Value	HR (95% CI)	*p*-Value	HR (95% CI)	*p*-Value
*BTLA*	rs9288952 (d)	1.08 (0.92–1.26)	0.353	1.11 (0.90–1.37)	0.309	1.06 (0.74–1.52)	0.744
*BTLA*	rs76844316 (d)	1.01 (0.78–1.30)	0.952	1.00 (0.76–1.32)	0.994	N.A. ^†^	N.A.
*PDCD1*	rs2227982 (d)	0.97 (0.83–1.12)	0.653	0.98 (0.77–1.24)	0.859	0.93 (0.73–1.19)	0.572
*LAG3*	rs870849 (d)	1.05 (0.85–1.30)	0.648	1.04 (0.82–1.31)	0.743	N.A. ^†^	N.A.
*CTLA4*	rs231775 (d)	0.95 (0.81–1.11)	0.505	0.91 (0.73–1.13)	0.403	0.98 (0.73–1.31)	0.872
*EZH2*	rs2302427 (d)	1.07 (0.83–1.37)	0.595	0.97 (0.73–1.29)	0.830	N.A. ^†^	N.A.
*DNMT1*	rs2228612 (d)	1.00 (0.85–1.18)	0.977	1.05 (0.85–1.30)	0.623	0.87 (0.60–1.25)	0.456
*BTLA*	rs9288952 (r)	1.00 (0.85–1.18)	0.975	1.06 (0.86–1.31)	0.602	0.81 (0.54–1.23)	0.328
*BTLA*	rs76844316 (r)	1.10 (0.85–1.44)	0.461	1.01 (0.75–1.35)	0.950	N.A. ^†^	N.A.
*PDCD1*	rs2227982 (r)	1.04 (0.90–1.21)	0.587	0.97 (0.77–1.23)	0.809	1.11 (0.87–1.41)	0.399
*LAG3*	rs870849 (r)	0.98 (0.81–1.19)	0.838	0.99 (0.79–1.24)	0.942	N.A. ^†^	N.A.
*CTLA4*	rs231775 (r)	1.10 (0.94–1.28)	0.227	1.18 (0.94–1.47)	0.146	1.01 (0.75–1.36)	0.954
*EZH2*	rs2302427 (r)	0.81 (0.61–1.09)	0.161	0.78 (0.57–1.07)	0.123	N.A. ^†^	N.A.
*DNMT1*	rs2228612 (r)	1.03 (0.89–1.20)	0.691	1.01 (0.82–1.25)	0.922	1.13 (0.84–1.52)	0.422

The results for each SNP were obtained by running separate regressions under the three genetic models, indicated in the top row of the table, adjusted with HLA-C mismatch, ABO mismatch, recipient age, donor age, PS, disease stage, and female donor–male recipient. Malignant-disease patients without a previous transplantation history were analyzed (*n* = 822). The number of primary events (death) = 354. ^†^ Not applicable (N.A.) due to a low minor allele frequency. HR stands for hazard ratio. See the legend of Table 1 for other notations. No SNP correlated with OS (*p* > 0.005 by the Wald test).

**Table 6:** Multivariable regression of non-relapse mortality (NRM) on donor and recipient SNPs.

Gene	SNP (Donor/Recipient)	Additive Model		Dominant Model		Recessive Model	
		SHR (95% CI)	*p*-Value	SHR (95% CI)	*p*-Value	SHR (95% CI)	*p*-Value
*BTLA*	rs9288952 (d)	1.07 (0.85–1.36)	0.541	1.04 (0.78–1.40)	0.786	1.27 (0.77–2.09)	0.344
*BTLA*	rs76844316 (d)	1.17 (0.82–1.67)	0.381	1.16 (0.80–1.69)	0.430	N.A. ^†^	N.A.
*PDCD1*	rs2227982 (d)	0.92 (0.74–1.12)	0.399	0.97 (0.69–1.35)	0.849	0.80 (0.55–1.16)	0.245
*LAG3*	rs870849 (d)	1.09 (0.81–1.46)	0.573	1.06 (0.76–1.46)	0.746	N.A. ^†^	N.A.
*CTLA4*	rs231775 (d)	0.93 (0.75–1.17)	0.539	0.82 (0.61–1.11)	0.203	1.10 (0.74–1.63)	0.634
*EZH2*	rs2302427 (d)	0.90 (0.60–1.35)	0.613	0.85 (0.55–1.31)	0.453	N.A. ^†^	N.A.
*DNMT1*	rs2228612 (d)	1.00 (0.80–1.25)	0.984	1.03 (0.77–1.39)	0.841	0.91 (0.55–1.51)	0.721
*BTLA*	rs9288952 (r)	1.01 (0.81–1.27)	0.897	1.04 (0.77–1.39)	0.805	0.96 (0.55–1.66)	0.882
*BTLA*	rs76844316 (r)	1.12 (0.75–1.66)	0.578	1.00 (0.66–1.51)	0.990	N.A. ^†^	N.A.
*PDCD1*	rs2227982 (r)	1.03 (0.83–1.26)	0.811	1.09 (0.77–1.53)	0.623	0.98 (0.69–1.39)	0.902
*LAG3*	rs870849 (r)	0.82 (0.62–1.09)	0.171	0.84 (0.60–1.17)	0.291	N.A. ^†^	N.A.
*CTLA4*	rs231775 (r)	1.10 (0.89–1.36)	0.383	1.20 (0.88–1.65)	0.244	1.02 (0.66–1.57)	0.930
*EZH2*	rs2302427 (r)	0.90 (0.59–1.36)	0.613	0.86 (0.55–1.36)	0.523	N.A. ^†^	N.A.
*DNMT1*	rs2228612 (r)	1.15 (0.93–1.40)	0.192	1.16 (0.86–1.57)	0.320	1.27 (0.87–1.86)	0.216

The results for each SNP were obtained by running separate regressions under the three genetic models, indicated in the top row of the table, adjusted with HLA-C mismatch, ABO mismatch, recipient age, donor age, PS, disease stage, and female donor–male recipient. Malignant-disease patients without a previous transplantation history were analyzed (*n* = 766). Excluded: no complete remission achieved after BMT (*n* = 56). The number of primary competing events (NRM) = 182. ^†^ Not applicable (N.A.) due to a low minor allele frequency. See the legend of Table 1 for other notations. No SNP correlated with NRM (*p* > 0.005 by the Wald test).

**Table 7:** Multivariable regression of relapse on donor and recipient SNPs.

Gene	SNP (Donor/Recipient)	Additive Model		Dominant Model		Recessive Model	
		SHR (95% CI)	*p*-Value	SHR (95% CI)	*p*-Value	SHR (95% CI)	*p*-Value
*BTLA*	rs9288952 (d)	1.02 (0.81–1.29)	0.876	1.09 (0.79–1.50)	0.594	0.83 (0.45–1.50)	0.529
*BTLA*	rs76844316 (d)	0.79 (0.51–1.23)	0.304	0.74 (0.47–1.16)	0.192	N.A. ^†^	N.A.
*PDCD1*	rs2227982 (d)	0.94 (0.75–1.17)	0.558	0.94 (0.65–1.36)	0.751	0.89 (0.61–1.29)	0.528
*LAG3*	rs870849 (d)	0.92 (0.67–1.27)	0.613	0.98 (0.68–1.41)	0.920	N.A. ^†^	N.A.
*CTLA4*	rs231775 (d)	1.03 (0.82–1.29)	0.802	1.20 (0.85–1.70)	0.306	0.80 (0.50–1.30)	0.375
*EZH2*	rs2302427 (d)	1.12 (0.76–1.67)	0.566	1.11 (0.73–1.69)	0.630	N.A. ^†^	N.A.
*DNMT1*	rs2228612 (d)	1.07 (0.84–1.38)	0.582	1.07 (0.77–1.48)	0.675	1.15 (0.68–1.94)	0.607
*BTLA*	rs9288952 (r)	0.95 (0.74–1.22)	0.674	0.92 (0.66–1.27)	0.601	0.98 (0.56–1.74)	0.955
*BTLA*	rs76844316 (r)	0.87 (0.56–1.35)	0.539	0.83 (0.51–1.34)	0.444	N.A. ^†^	N.A.
*PDCD1*	rs2227982 (r)	1.07 (0.86–1.34)	0.524	1.07 (0.74–1.53)	0.721	1.14 (0.80–1.62)	0.480
*LAG3*	rs870849 (r)	1.04 (0.77–1.41)	0.781	0.97 (0.68–1.38)	0.861	N.A. ^†^	N.A.
*CTLA4*	rs231775 (r)	0.97 (0.77–1.22)	0.766	1.00 (0.72–1.39)	1.00	0.87 (0.54–1.41)	0.570
*EZH2*	rs2302427 (r)	0.68 (0.43–1.07)	0.098	0.70 (0.43–1.15)	0.158	N.A. ^†^	N.A.
*DNMT1*	rs2228612 (r)	0.83 (0.64–1.07)	0.146	0.71 (0.52–0.99)	0.041	0.98 (0.61–1.59)	0.946

The results for each SNP were obtained by running separate regressions under the three genetic models, indicated in the top row of the table, adjusted with HLA-C mismatch, ABO mismatch, recipient age, donor age, PS, disease stage, and female donor–male recipient. Malignant-disease patients without a previous transplantation history were analyzed (*n* = 766). Excluded: no complete remission achieved after BMT (*n* = 56). The number of primary competing events (relapse) = 152. ^†^ Not applicable (N.A.) due to a low minor allele frequency. See the legend of Table 1 for other notations. No SNP correlated with relapse (*p* > 0.005 by the Wald test).

## Data Availability

The data presented in this study are available on request from the corresponding author.

## Supplementary Materials

The following are available online at https://www.mdpi.com/article/10.3390/cancers13112752/s1, Table S1. Characteristics of 822 malignant-disease patients without a transplantation history and their uBMT donor, Table S2. SNP information, Table S3. Summary of genotyping of 999 donors and 999 recipients, Table S4. SNP frequency, Table S5. Unphased linkage disequilibrium (LD) between SNPs, Table S6. Univariable subdistribution hazard (SH) regression of grade 2–4 acute GVHD (aGVHD), Table S7. Univariable SH regression of grade 3–4 aGVHD, Table S8. Univariable SH regression of extensive chronic GVHD (ecGVHD), Table S9. Univariable SH regression of all chronic GVHD (cGVHD), Table S10. Univariable Cox’s regression of overall survival (OS), Table S11. Univariable SH regression of non-relapse mortality (NRM), Table S12. Univariable SH regression of relapse.

## References

[1] Lee S.J., Klein J., Haagenson M., Baxter-Lowe L.A., Confer D.L., Eapen M., Fernandez-Vina M., Flomenberg N., Horowitz M., Hurley C.K. (2007). High-resolution donor-recipient HLA matching contributes to the success of unrelated donor marrow transplantation. Blood.

[2] Furst D., Muller C., Vucinic V., Bunjes D., Herr W., Gramatzki M., Schwerdtfeger R., Arnold R., Einsele H., Wulf G. (2013). High-resolution HLA matching in hematopoietic stem cell transplantation: A retrospective collaborative analysis. Blood.

[3] Morishima Y., Kashiwase K., Matsuo K., Azuma F., Morishima S., Onizuka M., Yabe T., Murata M., Doki N., Eto T. (2015). Biological significance of HLA locus matching in unrelated donor bone marrow transplantation. Blood.

[4] Dickinson A.M., Norden J. (2015). Non-HLA genomics: Does it have a role in predicting haematopoietic stem cell transplantation outcome?. Int. J. Immunogenet..

[5] Tanabe T., Yamaguchi N., Matsuda K., Yamazaki K., Takahashi S., Tojo A., Onizuka M., Eishi Y., Akiyama H., Ishikawa J. (2011). Association analysis of the NOD2 gene with susceptibility to graft-versus-host disease in a Japanese population. Int. J. Hematol..

[6] Takahashi H., Okayama N., Yamaguchi N., Miyahara Y., Morishima Y., Suehiro Y., Yamasaki T., Tamada K., Takahashi S., Tojo A. (2017). Associations of interactions between NLRP3 SNPs and HLA mismatch with acute and extensive chronic graft-versus-host diseases. Sci. Rep..

[7] Chambers C.A., Kuhns M.S., Egen J.G., Allison J.P. (2001). CTLA-4-mediated inhibition in regulation of T cell responses: Mechanisms and manipulation in tumor immunotherapy. Annu. Rev. Immunol..

[8] Iwai Y., Hamanishi J., Chamoto K., Honjo T. (2017). Cancer immunotherapies targeting the PD-1 signaling pathway. J. Biomed. Sci..

[9] Watanabe N., Gavrieli M., Sedy J.R., Yang J., Fallarino F., Loftin S.K., Hurchla M.A., Zimmerman N., Sim J., Zang X. (2003). BTLA is a lymphocyte inhibitory receptor with similarities to CTLA-4 and PD-1. Nat. Immunol..

[10] Wang J., Sanmamed M.F., Datar I., Su T.T., Ji L., Sun J., Chen L., Chen Y., Zhu G., Yin W. (2019). Fibrinogen-like Protein 1 Is a Major Immune Inhibitory Ligand of LAG-3. Cell.

[11] Sharma P., Allison J.P. (2020). Dissecting the mechanisms of immune checkpoint therapy. Nat. Rev. Immunol..

[12] Chamoto K., Hatae R., Honjo T. (2020). Current issues and perspectives in PD-1 blockade cancer immunotherapy. Int. J. Clin. Oncol..

[13] Zeiser R., Socie G., Blazar B.R. (2016). Pathogenesis of acute graft-versus-host disease: From intestinal microbiota alterations to donor T cell activation. Br. J. Haematol..

[14] Hu D., Shilatifard A. (2016). Epigenetics of hematopoiesis and hematological malignancies. Genes Dev..

[15] Wong K.K., Lawrie C.H., Green T.M. (2019). Oncogenic Roles and Inhibitors of DNMT1, DNMT3A, and DNMT3B in Acute Myeloid Leukaemia. Biomark. Insights.

[16] Orskov A.D., Treppendahl M.B., Skovbo A., Holm M.S., Friis L.S., Hokland M., Gronbaek K. (2015). Hypomethylation and up-regulation of PD-1 in T cells by azacytidine in MDS/AML patients: A rationale for combined targeting of PD-1 and DNA methylation. Oncotarget.

[17] Choi J., Ritchey J., Prior J.L., Holt M., Shannon W.D., Deych E., Piwnica-Worms D.R., DiPersio J.F. (2010). In vivo administration of hypomethylating agents mitigate graft-versus-host disease without sacrificing graft-versus-leukemia. Blood.

[18] Zhang W., Xu J. (2017). DNA methyltransferases and their roles in tumorigenesis. Biomark. Res..

[19] Cao R., Wang L., Wang H., Xia L., Erdjument-Bromage H., Tempst P., Jones R.S., Zhang Y. (2002). Role of histone H3 lysine 27 methylation in Polycomb-group silencing. Science.

[20] Vire E., Brenner C., Deplus R., Blanchon L., Fraga M., Didelot C., Morey L., Van Eynde A., Bernard D., Vanderwinden J.M. (2006). The Polycomb group protein EZH2 directly controls DNA methylation. Nature.

[21] Sashida G., Iwama A. (2017). Multifaceted role of the polycomb-group gene EZH2 in hematological malignancies. Int. J. Hematol..

[22] Nakagawa M., Kitabayashi I. (2018). Oncogenic roles of enhancer of zeste homolog 1/2 in hematological malignancies. Cancer Sci..

[23] He S., Xie F., Liu Y., Tong Q., Mochizuki K., Lapinski P.E., Mani R.S., Reddy P., Mochizuki I., Chinnaiyan A.M. (2013). The histone methyltransferase Ezh2 is a crucial epigenetic regulator of allogeneic T-cell responses mediating graft-versus-host disease. Blood.

[24] Alahmari B., Cooper M., Ziga E., Ritchey J., DiPersio J.F., Choi J. (2018). Selective targeting of histone modification fails to prevent graft versus host disease after hematopoietic cell transplantation. PLoS ONE.

[25] Burr M.L., Sparbier C.E., Chan K.L., Chan Y.C., Kersbergen A., Lam E.Y.N., Azidis-Yates E., Vassiliadis D., Bell C.C., Gilan O. (2019). An Evolutionarily Conserved Function of Polycomb Silences the MHC Class I Antigen Presentation Pathway and Enables Immune Evasion in Cancer. Cancer Cell.

[26] Perez-Garcia A., De la Camara R., Roman-Gomez J., Jimenez-Velasco A., Encuentra M., Nieto J.B., de la Rubia J., Urbano-Ispizua A., Brunet S., Iriondo A. (2007). CTLA-4 polymorphisms and clinical outcome after allogeneic stem cell transplantation from HLA-identical sibling donors. Blood.

[27] Piccioli P., Balbi G., Serra M., Morabito A., Lamparelli T., Gobbi M., Laurent S., Dozin B., Bruzzi P., Ferraris A.M. (2010). CTLA-4 +49A>G polymorphism of recipients of HLA-matched sibling allogeneic stem cell transplantation is associated with survival and relapse incidence. Ann. Hematol..

[28] Sellami M.H., Bani M., Torjemane L., Kaabi H., Ladeb S., Ben Othmane T., Hmida S. (2011). Effect of donor CTLA-4 alleles and haplotypes on graft-versus-host disease occurrence in Tunisian patients receiving a human leukocyte antigen-identical sibling hematopoietic stem cell transplant. Hum. Immunol..

[29] Chien J.W., Zhang X.C., Fan W., Wang H., Zhao L.P., Martin P.J., Storer B.E., Boeckh M., Warren E.H., Hansen J.A. (2012). Evaluation of published single nucleotide polymorphisms associated with acute GVHD. Blood.

[30] Harkensee C., Oka A., Onizuka M., Middleton P.G., Inoko H., Hirayasu K., Kashiwase K., Yabe T., Nakaoka H., Gennery A.R. (2012). Single nucleotide polymorphisms and outcome risk in unrelated mismatched hematopoietic stem cell transplantation: An exploration study. Blood.

[31] Karabon L., Markiewicz M., Partyka A., Pawlak-Adamska E., Tomkiewicz A., Dzierzak-Mietla M., Kyrcz-Krzemien S., Frydecka I. (2015). A CT60G>A polymorphism in the CTLA-4 gene of the recipient may confer susceptibility to acute graft versus host disease after allogeneic hematopoietic stem cell transplantation. Immunogenetics.

[32] Qin X.Y., Wang Y., Li G.X., Qin Y.Z., Wang F.R., Xu L.P., Chen H., Han W., Wang J.Z., Zhang X.H. (2016). CTLA-4 polymorphisms and haplotype correlate with survival in ALL after allogeneic stem cell transplantation from related HLA-haplotype-mismatched donor. J. Transl. Med..

[33] Martin P.J., Fan W., Storer B.E., Levine D.M., Zhao L.P., Warren E.H., Flowers M.E., Lee S.J., Carpenter P.A., Boeckh M. (2016). Replication of associations between genetic polymorphisms and chronic graft-versus-host disease. Blood.

[34] Santos N., Rodriguez-Romanos R., de la Camara R., Brunet S., Nieto J.B., Buno I., Martinez C., Jimenez-Velasco A., Vallejo C., Gonzalez M. (2018). PD-1 genotype of the donor is associated with acute graft-versus-host disease after HLA-identical sibling donor stem cell transplantation. Ann. Hematol..

[35] Tiercy J.M. (2014). HLA-C Incompatibilities in Allogeneic Unrelated Hematopoietic Stem Cell Transplantation. Front. Immunol..

[36] Saito H., Ito M., Kato S., Kodera Y., Okamoto S., Taniguchi S., Takanashi M., Kanamori H., Masaoka T., Takaku F. (2018). The Japan Marrow Donor Program, 25 years of experience in achieving 20,000 bone marrow transplantations: Organization structure, activity, and financial basis. Bone Marrow Transplant..

[37] Atsuta Y., Suzuki R., Yoshimi A., Gondo H., Tanaka J., Hiraoka A., Kato K., Tabuchi K., Tsuchida M., Morishima Y. (2007). Unification of hematopoietic stem cell transplantation registries in Japan and establishment of the TRUMP System. Int. J. Hematol..

[38] Auton A., Brooks L.D., Durbin R.M., Garrison E.P., Kang H.M., Korbel J.O., Marchini J.L., McCarthy S., McVean G.A., The 1000 Genomes Project Consortium (2015). A global reference for human genetic variation. Nature.

[39] Oki M., Watanabe N., Owada T., Oya Y., Ikeda K., Saito Y., Matsumura R., Seto Y., Iwamoto I., Nakajima H. (2011). A functional polymorphism in B and T lymphocyte attenuator is associated with susceptibility to rheumatoid arthritis. Clin. Dev. Immunol..

[40] Sun T., Zhou Y., Yang M., Hu Z., Tan W., Han X., Shi Y., Yao J., Guo Y., Yu D. (2008). Functional genetic variations in cytotoxic T-lymphocyte antigen 4 and susceptibility to multiple types of cancer. Cancer Res..

[41] Kanda Y. (2013). Investigation of the freely available easy-to-use software ‘EZR’ for medical statistics. Bone Marrow Transplant..

[42] Sakoda Y., Park J.J., Zhao Y., Kuramasu A., Geng D., Liu Y., Davila E., Tamada K. (2011). Dichotomous regulation of GVHD through bidirectional functions of the BTLA-HVEM pathway. Blood.

[43] Fu Z., Li D., Jiang W., Wang L., Zhang J., Xu F., Pang D., Li D. (2010). Association of BTLA gene polymorphisms with the risk of malignant breast cancer in Chinese women of Heilongjiang Province. Breast Cancer Res. Treat..

[44] Ansell S.M., Lesokhin A.M., Borrello I., Halwani A., Scott E.C., Gutierrez M., Schuster S.J., Millenson M.M., Cattry D., Freeman G.J. (2015). PD-1 blockade with nivolumab in relapsed or refractory Hodgkin’s lymphoma. N. Engl. J. Med..

[45] Blazar B.R., Carreno B.M., Panoskaltsis-Mortari A., Carter L., Iwai Y., Yagita H., Nishimura H., Taylor P.A. (2003). Blockade of programmed death-1 engagement accelerates graft-versus-host disease lethality by an IFN-gamma-dependent mechanism. J. Immunol..

[46] Gu Y., Xiao L., Gu W., Chen S., Feng Y., Wang J., Wang Z., Cai Y., Chen H., Xu X. (2018). Rs2227982 and rs2227981 in PDCD1 gene are functional SNPs associated with T1D risk in East Asian. Acta Diabetol..

[47] Qian B.P., Wang X.Q., Qiu Y., Jiang H., Ji M.L., Jiang J. (2013). An exon polymorphism of programmed cell death 1 gene is associated with both the susceptibility and thoracolumbar kyphosis severity of ankylosing spondylitis in a Chinese Han population. J. Orthop. Sci..

[48] Zhang Z., Duvefelt K., Svensson F., Masterman T., Jonasdottir G., Salter H., Emahazion T., Hellgren D., Falk G., Olsson T. (2005). Two genes encoding immune-regulatory molecules (LAG3 and IL7R) confer susceptibility to multiple sclerosis. Genes Immun..

[49] Sengsayadeth S., Wang T., Lee S.J., Haagenson M.D., Spellman S., Fernandez Vina M.A., Muller C.R., Verneris M.R., Savani B.N., Jagasia M. (2014). Cytotoxic T-lymphocyte antigen-4 single nucleotide polymorphisms are not associated with outcomes after unrelated donor transplantation: A center for international blood and marrow transplant research analysis. Biol. Blood Marrow Transplant..

[50] Li H., Liu J.W., Sun L.P., Yuan Y. (2017). A Meta-Analysis of the Association between DNMT1 Polymorphisms and Cancer Risk. Biomed Res. Int..

[51] Li H., Chang C., Shang Y., Qiang L., Zhang B., Bu B., Ren G., Song L., Shang M., Yu J. (2017). A missense variant in EZH2 is associated with colorectal cancer risk in a Chinese population. Oncotarget.

[52] Coccaro N., Zagaria A., Orsini P., Anelli L., Tota G., Casieri P., Impera L., Minervini A., Minervini C.F., Cumbo C. (2018). RARA and RARG gene downregulation associated with EZH2 mutation in acute promyelocytic-like morphology leukemia. Hum. Pathol..

