# Supplementary material for: Analysis of Relationships between Immune Checkpoint and Methylase Gene Polymorphisms and Outcomes after Unrelated Bone Marrow Transplantation
Source: Cancers (Basel). 2021 Jun 1;13(11):2752. doi: 10.3390/cancers13112752 (PMC8199545; doi:10.3390/cancers13112752)
Supplement: Supplementary file 1 [file cancers-13-02752-s001.zip › cancers-1219178-supplementary.pdf]

Supplementary Information for

Analysis of relationships between immune checkpoint and methylase gene polymorphisms and outcomes after unrelated bone marrow transplantation

Hidekazu Takahashi[1], Naoko Okayama[2], Natsu Yamaguchi[1], Moe Nomura[1], Yuta Miyahara[2], MH Mahbub[1], Ryosuke Hase[1], Yasuo Morishima[3], Yutaka Suehiro[4], Takahiro Yamasaki[2,4], Koji Tamada[5], Satoshi Takahashi[6], Arinobu Tojo[7] and Tsuyoshi Tanabe[1,*]

[1]Department of Public Health and Preventive Medicine, Yamaguchi University Graduate School of Medicine, Ube, Japan.

[2]Division of Laboratory, Yamaguchi University Hospital, Ube, Japan.

[3]Division of Epidemiology and Prevention, Aichi Cancer Center Research Institute, Nagoya, Japan.

[4]Department of Oncology and Laboratory Medicine, Yamaguchi University Graduate School of Medicine, Ube, Japan.

[5]Department of Immunology, Yamaguchi University Graduate School of Medicine, Ube, Japan.

[6]Department of Hematology and Oncology, Institute of Medical Science, The University of Tokyo, Tokyo, Japan.

[7]Project Professor, Tokyo Medical and Dental University, Tokyo, Japan.

[*]Correspondence: tanabe@yamaguchi-u.ac.jp.

Table of Contents

Content **Pages**

Table S1. Characteristics of 822 malignant-disease patients without a transplantation history and their uBMT donor 3

Table S2. SNP information 4

Table S3. Summary of genotyping of 999 donors and 999 recipients 5

Table S4. SNP frequency 6

Table S5. Unphased linkage disequilibrium (LD) between SNPs 7

Table S6. Univariable subdistribution hazard (SH) regression of grade 2–4 acute GVHD (aGVHD) 8

Table S7. Univariable SH regression of grade 3–4 aGVHD 9

Table S8. Univariable SH regression of extensive chronic GVHD (ecGVHD) 10

Table S9. Univariable SH regression of all chronic GVHD (cGVHD) 11

Table S10. Univariable Cox's regression of overall survival (OS) 12

Table S11. Univariable SH regression of non-relapse mortality (NRM) 13

Table S12. Univariable SH regression of relapse 14

Table S1. Characteristics of 822 malignant-disease patients without a transplantation history and their uBMT donor

**Recipient characteristics**	***N* = 822**
Sex	
Female	336
Male	486
Age (year)	
Low (1–44)	379
High (45–71)	443
Underlying disease	
Acute myeloid leukemia (AML)	342
Acute lymphoblastic leukemia (ALL)	178
Myelodysplastic syndromes (MDS)	120
Chronic myeloid leukemia (CML)	27
Other leukemia	66
Lymphoid malignancy (LM)	76
Myeloproliferative disorders (MPD)	8
Plasma cell dyscrasias (PCD)	3
Solid tumors (ST)	2
Disease stage	
Standard	450
Advanced or unknown	372
Body mass index (BMI) (kg/m^2)	
Low (12.0–22.0) or unknown	469
High (22.0–38.6)	353
CMV serostatus before BMT	
Negative	141
Positive or unknown	681
Performance status (PS) before BMT	
Low (0)	478
High (1–4)	344

**Donor characteristics**	***N* = 822**
Sex	
Female	260
Male	562
Age	
Low (20–33 years)	396
High (34–68 years)	426
CMV serostatus before BMT	
Negative	253
Positive or unknown	569

**uBMT characteristics**	***N* = 822**
Myeloablative conditioning	
No	177
Yes	645
Cyclosporine A (CyA)||	
No or unknown	597
Yes	225
Ara-C	
No	731
Yes	91
Cyclophosphamide	
No	354
Yes	468
N of nucleated cells infused¶	
Low (0.01-2.50) or unknown	430
High (≥2.50)	392
Days from diagnosis to BMT	
Low (9-269)	415
High (270-10897) or unknown	407
ABO blood type	
Match	464
Mismatch	358
HLA-C	
0 mismatches	636
1 mismatch (both directions)	147
1 mismatch (GvH direction)	9
1 mismatch (rejection direction)	16
2 mismatches (both directions)	14
HLA-DQB1	
0 mismatches	758
1 mismatch (both directions)	52
1 mismatch (GvH direction)	5
1 mismatch (rejection direction)	7
2 mismatches (both directions)	0
HLA-DPB1	
0 mismatches	195
1 mismatch (both directions)	331
1 mismatch (GvH direction)	68
1 mismatch (rejection direction)	68
2 mismatches (both directions)	160
HLA-C, -DQB1 or -DPB1 (GvH)	
0 mismatches	214
1 mismatch (GvH direction)	330
2 mismatches (GvH direction)	211
3 mismatches (GvH direction)	60
4 mismatches (GvH direction)	7
HLA-C, -DQB1 or -DPB1 (bi-directional)	
0 mismatches	155
1 mismatch (either direction)	369
2 mismatches (either direction)	223
3 mismatches (either direction)	64
4 mismatches (either direction)	11

These 822 pairs are identical to Group 1 described in Supplementary Table S1 of Takahashi et al.[6].

||Tacrolimus was administered to 594 out of the 596 CyA non-users and to 6 out of the 225 CyA users. ¶10^8 per kg body weight.

Table S2. SNP information

SNP	Alternative name	Gene	SNP type	Chromosome	Location in GRCh37*	Location in GRCh38*	Allele 1†	Allele 2†
rs9288952	+800C>T	*BTLA*	Non-synonymous (P219L)	3	112185025	112466178	A(Leu)	G(Pro)
rs76844316	+590A>C	*BTLA*	Non-synonymous (N149T)	3	112188609	112469762	T(Asn)	G(Thr)
rs2227982	PD-1.9 C/T	*PDCD1*	Non-synonymous (V215A)	2	242793433	241851281	G(Ala)	A(Val)
rs870849		*LAG3*	Non-synonymous (T455I)	12	6887020	6777854	C(Thr)	T(Ile)
rs231775	+49	*CTLA4*	Non-synonymous (T17A)	2	204732714	203867991	G(Ala)	A(Thr)
rs2302427		*EZH2*	Non-synonymous (D185H)	7	148525904	148828812	C(Asp)	G(His)
rs2228612		*DNMT1*	Non-synonymous (I327V)	19	10273372	10162696	T(Ile)	C(Val)

SNP	Reported molecular function of allele 2 relative to allele 1	DNA sequence around the SNP
rs9288952	-	GCCAGTCTTGAGTTC[**A/G**]GTCCAATGACAGAAT
rs76844316	The +590C, but not +590A, allele introduced into Jurkat T cells lacked inhibitory activity against the induction of IL-2[39]	ATCAAGCTTACCAGG[**G/T**]TAATTTCCCTTCCTG
rs2227982	-	GAGAACACAGGCACG[**A/G**]CTGAGGGGTCCTCCT
rs870849	-	TCCTGCTGTTTCTCA[**C/T**]CCTTGGTGTCCTTTC
rs231775	T cells with the AA genotype had lower activation. CTLA4-Thr17 more strongly binds to B7.1 and is a stronger inhibitor of T-cell activation[40]	CAGCTGAACCTGGCT[**A/G**]CCAGGACCTGGCCCT
rs2302427	-	CATCATCATCATCGT[**C/G**]ATCATCATTATATTG
rs2228612	-	CTTTTTCATCAGAAA[**C/T**]CTGTGGATTTACTTT

SNP	TaqMan Assay ID	PCR primer A (5' -> 3')	PCR primer B (5' -> 3')
rs9288952	C___1175845_10	CTGAAGTTTATTCTAATCCATGCCTGG	GACTTAACTCCTCACACATATGGATGC
rs76844316	C_100032809_10	GGCAAGGTGGTTTACTTCTGCTTTC	GCCTGGCACATGGTGTTATATAAATTC
rs2227982	C__57931287_10	CTCAGGGTAAGCAGCTCATAGTGG	CATAGTCCACAGAGAACACAGGCA
rs870849	C___9797874_10	GACAAAGTGTCCTTTCTAGTCCTGACC	CTGGCTCACCTGTCTTCTCCAAAG
rs231775	C___2415786_20	GCTCTACTTCCTGAAGACCTGAACAC	GTAGGAGAAACACCTCCTCCATCTTC
rs2302427	C__15757626_10	CTGACATTTGATGCGTTTCAGAATG	GAAAGCTGTAATGGCTACACAGAATCC
rs2228612	C__16171472_10	AGGCATTAGTTTGTCACTTTCCGTG	GTGAGACCTTTACCTTTTCATCCTCG

TaqMan assays and the oligo DNA sequences used for direct DNA sequencing are shown.

*GRCh37 and 38 stand for Genome Reference Consortium human genome build 37 and build 38, respectively.

†Alleles 1 and 2 exhibit variant nucleotides with corresponding amino acid residues in parentheses.

Table S3. Summary of genotyping of 999 donors and 999 recipients

	Gene	N of subjects successfully genotyped in one round of the TaqMan assay	N of subjects successfully genotyped by PCR direct sequencing after a failure in the TaqMan assay	N of subjects genotyped only by PCR direct sequencing	N of subjects with the final genotype undetermined
rs9288952	*BTLA*	1961	27	10	0
rs76844316	*BTLA*	1975	13	10	0
rs2227982	*PDCD1*	1950	38	10	0
rs870849	*LAG3*	1965	22	10	1
rs231775	*CTLA4*	1952	36	10	0
rs2302427	*EZH2*	1980	8	10	0
rs2228612	*DNMT1*	1970	18	10	0

Table S4. SNP frequency

	Donor		Recipient		1000 Genomes
	Malignant diseases	All diseases	Malignant diseases	All diseases	JPT104
N	822	887	822	887	104
rs9288952 (*BTLA*)					
AA	425	457	449	487	56
AG	329	354	306	329	44
GG	68	76	67	71	4
G allele frequency	0.283	0.285	0.268	0.266	0.25
P for HWE	0.731	0.511	0.155	0.144	0.294
rs76844316 (*BTLA*)					
TT	681	735	698	752	92
GT	132	143	116	125	11
GG	9	9	8	10	1
G allele frequency	0.091	0.091	0.08	0.082	0.063
P for HWE	0.396	0.421	0.23	0.073	0.329
rs2227982 (*PDCD1*)					
GG	204	215	229	252	30
AG	404	447	394	419	50
AA	214	225	199	216	24
A allele frequency	0.506	0.506	0.482	0.48	0.471
P for HWE	0.626	0.84	0.264	0.107	0.698
rs870849 (*LAG3*)					
CC	594	643	569	619	64
CT	211	227	226	241	40
TT	17	17	27	27	0
T allele frequency	0.149	0.147	0.17	0.166	0.192
P for HWE	0.89	0.688	0.458	0.545	0.012
rs231775 (*CTLA4*)					
GG	311	335	330	356	39
AG	384	419	373	406	52
AA	127	133	119	125	13
A allele frequency	0.388	0.386	0.372	0.37	0.375
P for HWE	0.659	0.944	0.412	0.614	0.675
rs2302427 (*EZH2*)					
CC	684	737	697	751	86
CG	126	138	116	125	16
GG	12	12	9	11	2
G allele frequency	0.091	0.091	0.082	0.083	0.096
P for HWE	0.035	0.068	0.102	0.042	0.229
rs2228612 (*DNMT1*)					
TT	374	402	374	398	50
CT	362	389	341	373	46
CC	86	96	107	116	8
C allele frequency	0.325	0.328	0.338	0.341	0.298
P for HWE	0.937	0.879	0.042	0.062	0.645

Genotype frequency and the Hardy–Weinberg equilibrium (HWE) of SNPs in malignant-disease first-time transplantation recipients and their donors (Supplementary Table S1), in all-disease first-time transplantation recipients and their donors (Groups 1+2 in Supplementary Table S1 of Takahashi et al.[6]), and in Japanese residents of Tokyo (JPT104) of 1000 Genomes Project are shown. Yellow highlighting indicates $P < 0.05$, although no significant ($P < 0.005$) violation of HWE was observed (exact test).

Table S5. Unphased linkage disequilibrium (LD) between SNPs

	Donor (N = 887)	Recipient (N = 887)	JPT104 (N = 104)
R^2 between rs9288952 and rs76844316	0.24*	0.24*	0.20*

The R^2 values among the two *BTLA* SNPs in all-disease first-time transplantation recipients (Groups 1+2 in Supplementary Table S1 of Takahashi et al.[6]), in their donors, and in JPT104 are shown. *P < 0.005 (the chi-squared test). As expected from distant genomic locations, no other pairwise combinations of the eight SNPs analyzed in the present study exhibited R^2 > 0.02.

Table S6. Univariable subdistribution hazard (SH) regression of grade 2–4 acute GVHD (aGVHD)

Gene	SNP (donor /recipient)	Additive model		Dominant model		Recessive model	
		SHR (95% CI)	*P*	SHR (95% CI)	*P*	SHR (95% CI)	*P*
BTLA	rs9288952 (d)	1.06 (0.88–1.27)	.531	1.10 (0.87–1.39)	.409	0.99 (0.63–1.55)	.964
BTLA	rs76844316 (d)	1.00 (0.76–1.31)	.985	1.06 (0.78–1.46)	.706	N.A.†	N.A.
PDCD1	rs2227982 (d)	0.95 (0.80–1.12)	.537	0.89 (0.68–1.16)	.379	0.98 (0.75–1.28)	.887
LAG3	rs870849 (d)	1.00 (0.80–1.26)	.997	1.02 (0.79–1.32)	.863	N.A.†	N.A.
CTLA4	rs231775 (d)	0.86 (0.72–1.01)	.070	0.82 (0.65–1.04)	.103	0.80 (0.57–1.12)	.197
EZH2	rs2302427 (d)	0.80 (0.61–1.06)	.122	0.86 (0.63–1.19)	.368	N.A.†	N.A.
DNMT1	rs2228612 (d)	0.90 (0.75–1.08)	.259	0.92 (0.73–1.16)	.462	0.77 (0.49–1.19)	.231
BTLA	rs9288952 (r)	0.87 (0.72–1.06)	.166	0.83 (0.65–1.05)	.121	0.90 (0.58–1.40)	.642
BTLA	rs76844316 (r)	0.88 (0.62–1.25)	.473‡	0.84 (0.58–1.20)	.331‡	N.A.†	N.A.
PDCD1	rs2227982 (r)	1.00 (0.85–1.17)	.989	1.05 (0.81–1.36)	.699	0.94 (0.71–1.25)	.671
LAG3	rs870849 (r)	0.98 (0.79–1.21)	.844	1.01 (0.78–1.29)	.954	N.A.†	N.A.
CTLA4	rs231775 (r)	0.97 (0.81–1.15)	.695	0.91 (0.72–1.15)	.440	1.05 (0.76–1.46)	.774
EZH2	rs2302427 (r)	0.65 (0.46–0.90)	.011	0.67 (0.46–0.96)	.030	N.A.†	N.A.
DNMT1	rs2228612 (r)	1.12 (0.95–1.33)	.173	1.09 (0.86–1.38)	.471	1.33 (0.97–1.82)	.073

The results for each SNP were obtained by running separate regressions under the three genetic models, indicated in the top row of the table. Malignant-disease patients without a previous transplantation history were analyzed (N = 787). Excluded: aGVHD-unevaluable (N = 34) and the day of grade 2/3/4 aGVHD unknown (N = 1). N of primary competing events (grade 2–4 aGVHD) = 280. No SNP correlated with grade 2–4 aGVHD ($P > 0.005$ by the Wald test). †Not applicable (N.A.) due to a low minor allele frequency. ‡The proportional SHR assumption is violated (P for the interaction between a variable and time < 0.005). See the legend of Table 1 for other notations.

Table S7. Univariable SH regression of grade 3–4 aGVHD

Gene	SNP (donor /recipient)	Additive model		Dominant model		Recessive model	
		SHR (95% CI)	*P*	SHR (95% CI)	*P*	SHR (95% CI)	*P*
BTLA	rs9288952 (d)	1.14 (0.81–1.60)	.466	1.09 (0.70–1.68)	.708	1.46 (0.73–2.90)	.280
BTLA	rs76844316 (d)	0.75 (0.42–1.32)	.315	0.77 (0.41–1.46)	.427	N.A.†	N.A.
PDCD1	rs2227982 (d)	0.88 (0.65–1.19)	.404	0.89 (0.55–1.45)	.636	0.79 (0.46–1.35)	.390
LAG3	rs870849 (d)	0.67 (0.39–1.14)	.138	0.59 (0.34–1.04)	.067	N.A.†	N.A.
CTLA4	rs231775 (d)	1.30 (0.97–1.74)	.081	1.61 (0.98–2.64)	.058	1.19 (0.67–2.10)	.554
EZH2	rs2302427 (d)	0.81 (0.48–1.37)	.429	0.86 (0.47–1.58)	.629	N.A.†	N.A.
DNMT1	rs2228612 (d)	1.10 (0.81–1.51)	.542	1.21 (0.78–1.89)	.403	0.96 (0.47–1.99)	.921
BTLA	rs9288952 (r)	0.89 (0.63–1.26)	.521	0.90 (0.58–1.40)	.641	0.75 (0.30–1.85)	.528
BTLA	rs76844316 (r)	0.87 (0.43–1.76)	.698	0.72 (0.36–1.45)	.358	N.A.†	N.A.
PDCD1	rs2227982 (r)	0.92 (0.69–1.24)	.591	0.97 (0.60–1.57)	.907	0.81 (0.47–1.40)	.452
LAG3	rs870849 (r)	0.88 (0.60–1.31)	.535	0.95 (0.59–1.52)	.816	N.A.†	N.A.
CTLA4	rs231775 (r)	1.03 (0.75–1.42)	.836	1.04 (0.67–1.64)	.850	1.05 (0.56–1.94)	.885
EZH2	rs2302427 (r)	0.75 (0.41–1.35)	.332	0.78 (0.40–1.50)	.451	N.A.†	N.A.
DNMT1	rs2228612 (r)	1.20 (0.87–1.66)	.275	1.12 (0.72–1.74)	.622	1.59 (0.91–2.79)	.106

The results for each SNP were obtained by running separate regressions under the three genetic models, indicated in the top row of the table. Malignant-disease patients without a previous transplantation history were analyzed (N = 787). Excluded: aGVHD-unevaluable (N = 34) and the day of grade 2/3/4 aGVHD unknown (N = 1). The number of primary competing events (grade 3–4 aGVHD) = 80. See the legend of Table 1 for other notations. No SNP correlated with grade 3–4 aGVHD ($P > 0.005$ by the Wald test).

Table S8. Univariable SH regression of extensive chronic GVHD (ecGVHD)

Gene	SNP (donor /recipient)	Additive model		Dominant model		Recessive model	
		SHR (95% CI)	*P*	SHR (95% CI)	*P*	SHR (95% CI)	*P*
BTLA	rs9288952 (d)	0.97 (0.74–1.27)	.822	0.94 (0.66–1.32)	.700	1.06 (0.57–1.95)	.859
BTLA	rs76844316 (d)	0.96 (0.66–1.42)	.848	1.05 (0.67–1.64)	.830	N.A.†	N.A.
PDCD1	rs2227982 (d)	1.10 (0.86–1.41)	.453	1.07 (0.71–1.60)	.760	1.19 (0.82–1.73)	.350
LAG3	rs870849 (d)	0.85 (0.59–1.22)	.377	0.84 (0.56–1.25)	.391	N.A.†	N.A.
CTLA4	rs231775 (d)	0.93 (0.72–1.19)	.559	0.90 (0.64–1.28)	.562	0.92 (0.57–1.48)	.728
EZH2	rs2302427 (d)	0.65 (0.40–1.07)	.092	0.63 (0.37–1.06)	.083	N.A.†	N.A.
DNMT1	rs2228612 (d)	1.08 (0.82–1.41)	.580	1.03 (0.73–1.45)	.865	1.30 (0.76–2.20)	.338
BTLA	rs9288952 (r)	0.88 (0.67–1.16)	.382	0.84 (0.60–1.20)	.344	0.89 (0.47–1.69)	.721
BTLA	rs76844316 (r)	1.28 (0.82–1.98)	.281	1.23 (0.78–1.96)	.377	N.A.†	N.A.
PDCD1	rs2227982 (r)	0.96 (0.76–1.22)	.746	0.98 (0.68–1.43)	.925	0.91 (0.60–1.38)	.658
LAG3	rs870849 (r)	0.77 (0.54–1.10)	.148	0.73 (0.49–1.08)	.112	N.A.†	N.A.
CTLA4	rs231775 (r)	0.88 (0.68–1.14)	.330	0.85 (0.61–1.20)	.367	0.84 (0.49–1.42)	.509
EZH2	rs2302427 (r)	1.08 (0.70–1.68)	.717	1.05 (0.65–1.69)	.841	N.A.†	N.A.
DNMT1	rs2228612 (r)	1.02 (0.80–1.30)	.883	1.05 (0.75–1.48)	.766	0.96 (0.57–1.63)	.886

The results for each SNP were obtained by running separate regressions under the three genetic models, indicated in the top row of the table. Malignant disease patients without a previous transplantation history were analyzed (N = 677). Excluded: cGVHD-unevaluable (N = 142) and the day of cGVHD unknown (N = 3). The number of primary competing events (ecGVHD) = 132. See the legend of Table 1 for other notations. No SNP correlated with ecGVHD ($P > 0.005$ by the Wald test).

Table S9. Univariable SH regression of all chronic GVHD (cGVHD)

Gene	SNP (donor /recipient)	Additive model		Dominant model		Recessive model	
		SHR (95% CI)	*P*	SHR (95% CI)	*P*	SHR (95% CI)	*P*
BTLA	rs9288952 (d)	0.95 (0.77–1.16)	.585	0.95 (0.73–1.22)	.676	0.88 (0.54–1.43)	.612
BTLA	rs76844316 (d)	1.17 (0.88–1.56)	.272	1.26 (0.91–1.74)	.167	N.A.†	N.A.
PDCD1	rs2227982 (d)	0.99 (0.83–1.19)	.935	1.02 (0.75–1.37)	.914	0.97 (0.72–1.30)	.814
LAG3	rs870849 (d)	0.94 (0.71–1.24)	.663	0.87 (0.64–1.16)	.338	N.A.†	N.A.
CTLA4	rs231775 (d)	1.00 (0.83–1.21)	.998	0.95 (0.73–1.24)	.718	1.09 (0.77–1.54)	.619
EZH2	rs2302427 (d)	0.70 (0.50–0.99)	.042	0.70 (0.48–1.03)	.068	N.A.†	N.A.
DNMT1	rs2228612 (d)	1.02 (0.84–1.24)	.843	1.04 (0.80–1.34)	.780	0.99 (0.64–1.54)	.978
BTLA	rs9288952 (r)	0.96 (0.79–1.17)	.698	0.99 (0.76–1.28)	.933	0.83 (0.51–1.36)	.460
BTLA	rs76844316 (r)	1.21 (0.86–1.69)	.276	1.22 (0.85–1.74)	.278	N.A.†	N.A.
PDCD1	rs2227982 (r)	0.94 (0.78–1.14)	.538	0.86 (0.65–1.13)	.276	1.01 (0.75–1.37)	.930
LAG3	rs870849 (r)	0.92 (0.72–1.17)	.491	0.89 (0.67–1.17)	.397	N.A.†	N.A.
CTLA4	rs231775 (r)	0.86 (0.72–1.04)	.125	0.87 (0.67–1.12)	.285	0.73 (0.48–1.11)	.138
EZH2	rs2302427 (r)	0.95 (0.68–1.33)	.768	0.95 (0.66–1.37)	.784	N.A.†	N.A.
DNMT1	rs2228612 (r)	0.92 (0.77–1.12)	.416	0.92 (0.71–1.19)	.534	0.85 (0.56–1.29)	.456

The results for each SNP were obtained by running separate regressions under the three genetic models, indicated in the top row of the table. Malignant-disease patients without a previous transplantation history were analyzed (N = 677). Excluded: cGVHD-unevaluable (N = 142) and the day of cGVHD unknown (N = 3). The number of primary competing events (limited + extensive cGVHD) = 235. †Not applicable (N.A.) due to a low minor allele frequency. See the legend of Table 1 for other notations. No SNP correlated with all cGVHD ($P > 0.005$ by the Wald test).

Table S10. Univariable Cox's regression of overall survival (OS)

Gene	SNP (donor /recipient)	Additive model		Dominant model		Recessive model	
		HR (95% CI)	*P*	HR (95% CI)	*P*	HR (95% CI)	*P*
BTLA	rs9288952 (d)	1.14 (0.97–1.34)	.106	1.17 (0.95–1.45)	.132	1.21 (0.84–1.73)	.307
BTLA	rs76844316 (d)	1.05 (0.82–1.35)	.697	1.08 (0.82–1.41)	.594	N.A.†	N.A.
PDCD1	rs2227982 (d)	0.91 (0.78–1.05)	.197	0.89 (0.70–1.12)	.314	0.87 (0.68–1.11)	.269
LAG3	rs870849 (d)	1.06 (0.86–1.30)	.604	1.07 (0.85–1.35)	.549	N.A.†	N.A.
CTLA4	rs231775 (d)	0.98 (0.84–1.14)	.809	0.97 (0.78–1.21)	.803	0.98 (0.73–1.31)	.898
EZH2	rs2302427 (d)	1.08 (0.85–1.39)	.518	0.99 (0.75–1.31)	.934	N.A.†	N.A.
DNMT1	rs2228612 (d)	1.02 (0.88–1.20)	.768	1.10 (0.89–1.36)	.357	0.86 (0.60–1.22)	.395
BTLA	rs9288952 (r)	1.01 (0.86–1.18)	.914	1.10 (0.89–1.35)	.377	0.76 (0.50–1.15)	.193
BTLA	rs76844316 (r)	1.08 (0.83–1.40)	.588	1.01 (0.76–1.35)	.935	N.A.†	N.A.
PDCD1	rs2227982 (r)	1.05 (0.91–1.21)	.501	1.04 (0.82–1.31)	.742	1.10 (0.87–1.40)	.430
LAG3	rs870849 (r)	0.99 (0.82–1.20)	.900	0.99 (0.79–1.24)	.945	N.A.†	N.A.
CTLA4	rs231775 (r)	1.12 (0.96–1.30)	.144	1.23 (0.99–1.52)	.065	1.05 (0.78–1.40)	.767
EZH2	rs2302427 (r)	0.79 (0.59–1.05)	.108	0.77 (0.56–1.05)	.098	N.A.†	N.A.
DNMT1	rs2228612 (r)	1.02 (0.88–1.19)	.783	0.99 (0.80–1.21)	.889	1.12 (0.84–1.51)	.440

The results for each SNP were obtained by running separate regressions under the three genetic models, indicated in the top row of the table. Malignant-disease patients without a previous transplantation history were analyzed (N = 822). The number of primary events (death) = 354. See the legend of Table 1 for other notations. No SNP correlated with OS ($P > 0.005$ by the Wald test).

Table S11. Univariable SH regression of non-relapse mortality (NRM)

Gene	SNP (donor /recipient)	Additive model		Dominant model		Recessive model	
		SHR (95% CI)	*P*	SHR (95% CI)	*P*	SHR (95% CI)	*P*
BTLA	rs9288952 (d)	1.12 (0.89–1.41)	.328	1.09 (0.82–1.46)	.541	1.36 (0.83–2.24)	.225
BTLA	rs76844316 (d)	1.22 (0.88–1.70)	.231	1.26 (0.88–1.81)	.206	N.A.†	N.A.
PDCD1	rs2227982 (d)	0.87 (0.71–1.06)	.160	0.92 (0.66–1.27)	.602	0.73 (0.51–1.05)	.092
LAG3	rs870849 (d)	1.10 (0.83–1.47)	.500	1.09 (0.80–1.50)	.579	N.A.†	N.A.
CTLA4	rs231775 (d)	0.94 (0.75–1.17)	.572	0.84 (0.63–1.13)	.250	1.09 (0.73–1.61)	.686
EZH2	rs2302427 (d)	0.89 (0.60–1.32)	.567	0.83 (0.54–1.25)	.365	N.A.†	N.A.
DNMT1	rs2228612 (d)	1.03 (0.83–1.27)	.815	1.07 (0.80–1.44)	.639	0.94 (0.58–1.52)	.791
BTLA	rs9288952 (r)	0.99 (0.79–1.23)	.905	1.01 (0.76–1.36)	.920	0.88 (0.51–1.52)	.653
BTLA	rs76844316 (r)	1.05 (0.71–1.55)	.803	0.95 (0.63–1.43)	.793	N.A.†	N.A.
PDCD1	rs2227982 (r)	1.02 (0.83–1.25)	.854	1.10 (0.79–1.54)	.558	0.95 (0.67–1.34)	.755
LAG3	rs870849 (r)	0.86 (0.65–1.13)	.276	0.87 (0.63–1.21)	.406	N.A.†	N.A.
CTLA4	rs231775 (r)	1.11 (0.91–1.36)	.315	1.23 (0.91–1.66)	.185	1.02 (0.67–1.55)	.916
EZH2	rs2302427 (r)	0.85 (0.56–1.29)	.443	0.81 (0.52–1.27)	.362	N.A.†	N.A.
DNMT1	rs2228612 (r)	1.17 (0.95–1.43)	.137	1.17 (0.87–1.57)	.290	1.35 (0.92–1.97)	.128

The results for each SNP were obtained by running separate regressions under the three genetic models indicated. Malignant-disease patients without a previous transplantation history were analyzed (N = 766). Excluded: no complete remission achieved after BMT (N = 56). The number of primary competing events (NRM) = 182. See the legend of Table 1 for other notations. No SNP correlated with NRM (*P* > 0.005 by the Wald test).

Table S12. Univariable SH regression of relapse

Gene	SNP (donor /recipient)	Additive model		Dominant model		Recessive model	
		SHR (95% CI)	*P*	SHR (95% CI)	*P*	SHR (95% CI)	*P*
BTLA	rs9288952 (d)	1.04 (0.83–1.32)	.729	1.12 (0.81–1.54)	.489	0.86 (0.48–1.55)	.614
BTLA	rs76844316 (d)	0.83 (0.54–1.28)	.408	0.79 (0.50–1.24)	.301	N.A.†	N.A.
PDCD1	rs2227982 (d)	0.93 (0.74–1.16)	.510	0.92 (0.64–1.31)	.633	0.89 (0.62–1.30)	.551
LAG3	rs870849 (d)	0.91 (0.66–1.24)	.546	0.98 (0.68–1.40)	.900	N.A.†	N.A.
CTLA4	rs231775 (d)	1.06 (0.86–1.31)	.590	1.28 (0.91–1.80)	.148	0.80 (0.50–1.28)	.349
EZH2	rs2302427 (d)	1.12 (0.77–1.65)	.549	1.13 (0.74–1.72)	.576	N.A.†	N.A.
DNMT1	rs2228612 (d)	1.07 (0.84–1.35)	.582	1.09 (0.79–1.50)	.604	1.09 (0.66–1.81)	.732
BTLA	rs9288952 (r)	0.97 (0.75–1.24)	.799	0.95 (0.69–1.31)	.736	1.00 (0.57–1.77)	.996
BTLA	rs76844316 (r)	0.92 (0.59–1.42)	.693	0.87 (0.54–1.40)	.569	N.A.†	N.A.
PDCD1	rs2227982 (r)	1.09 (0.88–1.36)	.439	1.09 (0.76–1.57)	.622	1.15 (0.81–1.65)	.428
LAG3	rs870849 (r)	1.04 (0.77–1.40)	.789	0.97 (0.69–1.37)	.855	N.A.†	N.A.
CTLA4	rs231775 (r)	0.97 (0.78–1.22)	.821	1.02 (0.74–1.41)	.906	0.87 (0.54–1.39)	.554
EZH2	rs2302427 (r)	0.72 (0.46–1.12)	.143	0.75 (0.46–1.22)	.244	N.A.†	N.A.
DNMT1	rs2228612 (r)	0.82 (0.64–1.05)	.121	0.71 (0.52–0.98)	.035	0.95 (0.59–1.53)	.833

The results for each SNP were obtained by running separate regressions under the three genetic models indicated. Malignant-disease patients without a previous transplantation history were analyzed (N = 766). Excluded: no complete remission achieved after BMT (N = 56). The number of primary competing events (relapse) = 152. See the legend of Table 1 for other notations. No SNP correlated with relapse ($P > 0.005$ by the Wald test).
